# Metasurface-enhanced mid-infrared spectroscopy in the liquid phase†

**DOI:** 10.1039/d2sc03927c

**Published:** 2022-10-21

**Authors:** Soheila Kharratian, Donato Conteduca, Barbara Procacci, Daniel J. Shaw, Neil T. Hunt, Thomas F. Krauss

**Affiliations:** Department of Chemistry and York Biomedical Institute, University of York Heslington York YO10 5DD UK soheila.kharratian@york.ac.uk; School of Physics, Engineering and Technology and York Biomedical Research Institute, University of York Heslington York YO10 5DD UK

## Abstract

Vibrational spectroscopy is an important tool in chemical and biological analysis. A key issue when applying vibrational spectroscopy to dilute liquid samples is the inherently low sensitivity caused by short interaction lengths and small extinction coefficients, combined with low target molecule concentrations. Here, we introduce a novel type of surface-enhanced infrared absorption spectroscopy based on the resonance of a dielectric metasurface. We demonstrate that the method is suitable for probing vibrational bands of dilute analytes with a range of spectral linewidths. We observe that the absorption signal is enhanced by 1–2 orders of magnitude and show that this enhancement leads to a lower limit of detection compared to attenuated total reflection (ATR). Overall, the technique provides an important addition to the spectroscopist's toolkit especially for probing dilute samples.

## Abstract 2

A novel surface-enhanced infrared absorption (SEIRA) spectroscopy method exploits the resonance of a dielectric metasurface to provide strong field enhancement at a microscale depth, making it suitable for analysis of dilute liquid samples.

## Graphical abstract

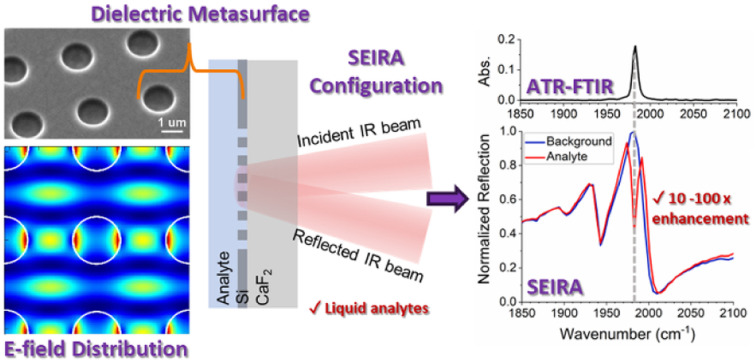

## Introduction

Vibrational absorption spectroscopy provides a powerful method for the label-free detection and investigation of molecular structures. The technique exploits characteristic vibrational absorption bands in the mid-infrared (mid-IR) spectrum to identify the chemical bonds and functional groups present in a molecule. Its chemical specificity is ideal for studying biological species and complex mixtures, yet its sensitivity is somewhat limited by the mismatch between the magnitude of the mid-IR wavelengths (2.5–15 μm) and the molecular dimensions, which results in small absorption cross-sections.^1–4^ Hence, concentrated samples or large optical path lengths (leading to large sample volumes) are typically required to produce sufficient signal/noise ratios, which limits the application of vibrational spectroscopy in a number of potentially important fields including clinical, biochemical and pharmaceutical applications.

Surface-enhanced infrared absorption (SEIRA) aims to address these limitations by improving the light–matter interaction, using plasmonic or photonic near-field enhancement. Plasmonic approaches provide strong localization of the electric field within nanometric volumes and significant enhancements within the plasmonic hotspot have been reported.^1,3,5–12^ Because of the nanometric distribution of the enhanced field, this approach is best suited to thin-film samples or surface-adsorbed molecules. Moreover, the intrinsic damping in metals imposes resistive losses to the plasmonic systems, which limits the quality of the resonance and diminishes the SEIRA signal.^4,6,13^ In contrast, photonic structures such as dielectric metasurfaces involve low losses, support resonances of higher quality and amplitude, and provide field enhancement at the microscale, which makes them much more suitable for investigating liquid samples, as are commonly found in biological analytes. Such metasurfaces have been demonstrated for SEIRA in physisorbed monolayers,^4,13,14^ but demonstrations in dilute liquid samples are yet to be reported.

Here, we demonstrate a Si-based all-dielectric metasurface that resonantly enhances the vibrational absorption of dilute liquid samples, enabling SEIRA analysis of solutions and opening the door to applications in biofluids. The resonance frequency of the metasurface can be adjusted *via* its periodicity, thereby tuning it to a specific molecular absorption band of the target analyte. We then show that the coupling of the molecular vibrations to the metasurface resonance enhances the absorption signal by 1–2 orders of magnitude. We demonstrate this photonic approach for SEIRA spectroscopy for both narrow and broad absorption bands. Furthermore, as the method works in reflection, our approach provides a route to overcoming potential challenges related to the obstructive solvent absorption, *e.g.* in aqueous solutions, by limiting the penetration depth of light.

## Results and discussion

**Fig. 1:**
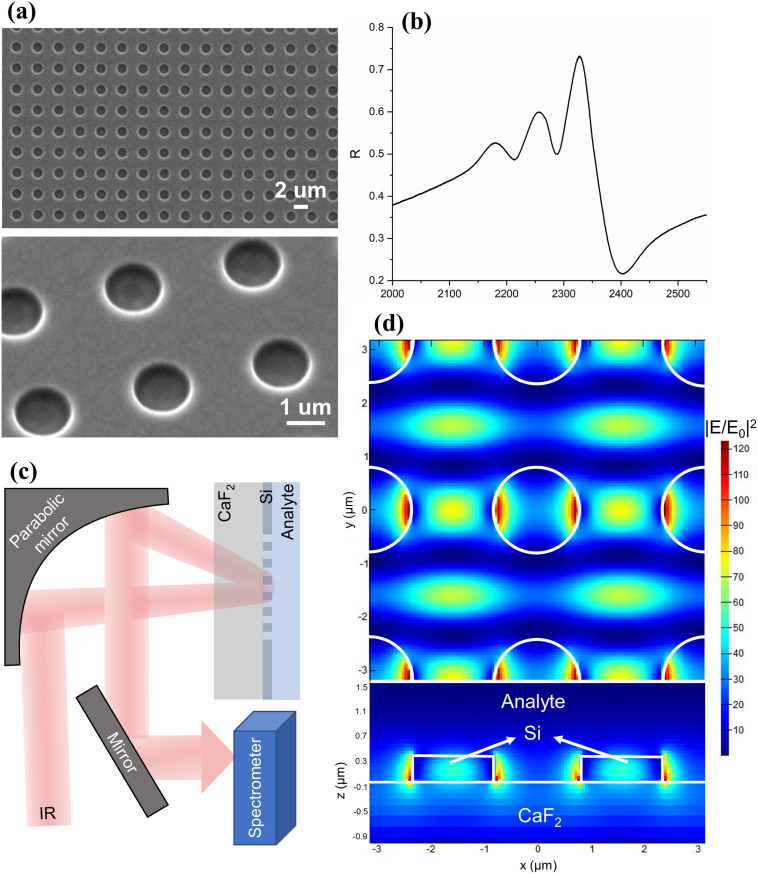
(a) Scanning electron micrographs of the metasurface, top and tilted view. The square array of holes is realized in a 400 nm thick amorphous Si layer on a CaF_2_ substrate. (b) The optical response of the metasurface showing an asymmetric Fano resonance. Reflection has been derived from Fourier transform infrared (FTIR) transmission measurements. (c) Schematic illustration of the experimental setup for the reflection-mode metasurface-enhanced absorption measurements. (d) Distribution of the enhanced electric field intensity at the resonance, top and cross-section view. *E* and *E*_0_ denote the local and incident electric fields, respectively, calculated using the finite-difference time-domain (FDTD) method.

The metasurface is comprised of a square array of holes etched into a 400 nm thick amorphous silicon layer on an IR-transparent CaF_2_ substrate. Scanning electron micrographs are shown in Fig. 1(a). Fig. 1(b) presents the optical response, measured by a Fourier transform IR (FTIR) spectrometer. The high-index Si layer performs both the grating diffraction and the waveguiding functions, thereby supporting guided-mode resonances (GMRs).^15^ The GMR can also be understood as an interference between the Fabry–Perot resonance of the thin film and the Bragg resonance of the array, leading to an asymmetric Fano lineshape.^16–19^ In fact, the spectrum shown here exhibits a slight deviation from the perfect Fano lineshape, in the spectral region around 2200–2300 cm^−1^, which is caused by the non-normal incidence angle used in our setup (Fig. 1(c) and S1†). Such angle-induced mode splitting is a well-understood phenomenon that has previously been reported.^20^ The thickness of the Si slab was chosen to maximise performance in terms of the amplitude and quality factor^14^ while ensuring ease of fabrication (see Fig. S2 in the ESI†).

Fig. 1(d) shows the spatial distribution of the near-field intensity of the resonance, obtained by finite-difference time-domain (FDTD) calculations, where *E* and *E*_0_ represent the local and incident electric fields, respectively. The simulation highlights an intensity enhancement greater than two orders of magnitude, which drives the enhanced light–matter interaction. Note that the field enhancement occurs over a depth of approximately 0.5 μm into the analyte (Fig. 1(c)), which is orders of magnitude larger than that for plasmonic SEIRA, making the technique more suitable for measurements in the liquid phase.

We now present two applications of metasurface enhancement, which differ in terms of the relationship between the linewidth of the molecular vibrational band and that of the metasurface resonance.

### Enhancement of narrow vibrational bands

**Fig. 2:**
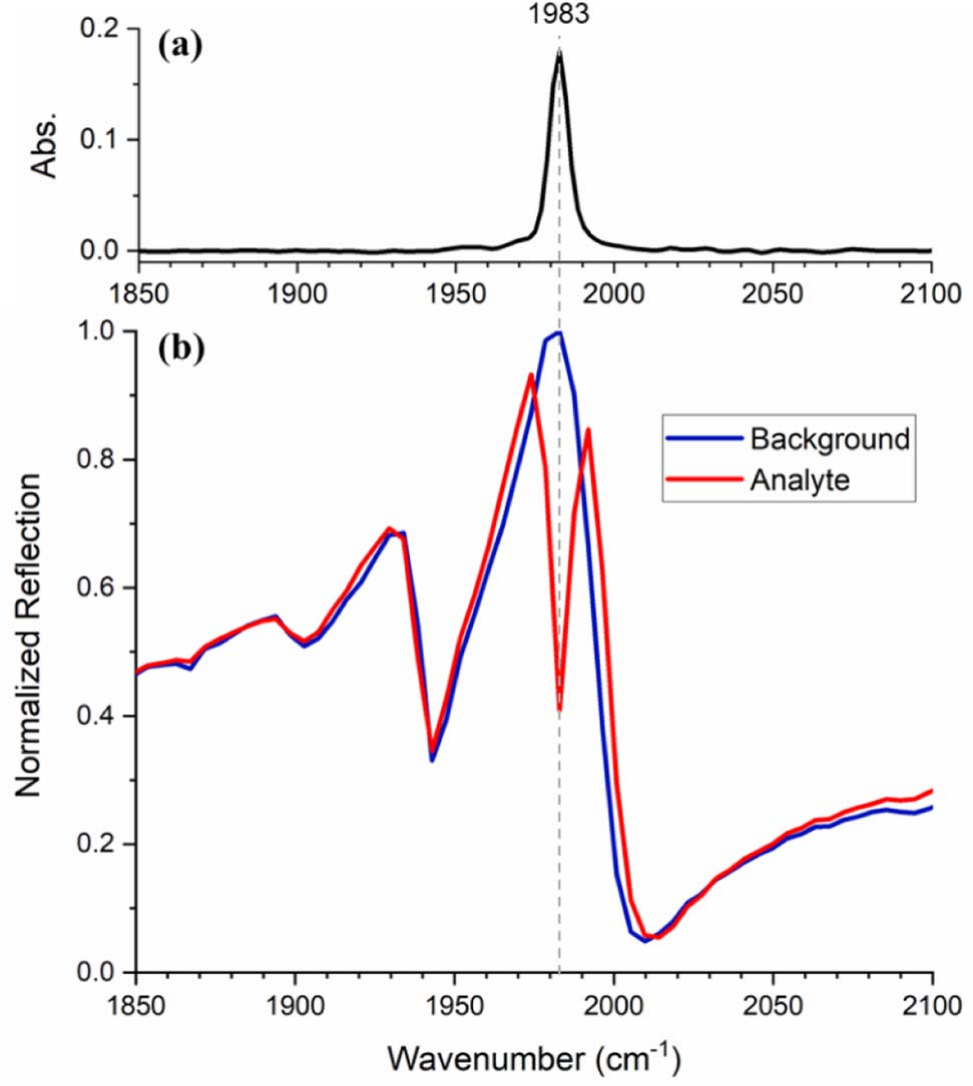
(a) Attenuated total reflection (ATR) spectrum of W(CO)_6_. (b) Normalized reflection spectra of the metasurface with heptane (blue) and W(CO)_6_/heptane solution (red).

First, we demonstrate the resonant absorption enhancement of an absorption band, which has a full width at half maximum (FWHM) smaller than that of the metasurface resonance. We use a 20 mM solution of W(CO)_6_ in heptane that exhibits a band in the IR spectrum at 1983 cm^−1^ with a FWHM of 7 cm^−1^ (Fig. 2(a)), assignable to the T_1u_ carbonyl stretching vibrational mode. A metasurface with a 3.171 (±0.001) μm period was fabricated to create a resonance matching this line. Fig. 2(b) shows the reflection spectrum of the metasurface in the presence of W(CO)_6_/heptane solution (red) in comparison with that of pure heptane (blue). Since the resonance of a bare metasurface would be shifted with respect to that in the presence of a liquid, the solvent spectrum is used as a reference to show the metasurface response only. The normalised reflection spectrum of the metasurface exhibits a clear Fano resonance with a peak and dip at 1983 and 2010 cm^−1^, respectively. The other dips around 1900 and 1940 cm^−1^ are due to the angled incidence, as discussed above. The FWHM of the Fano resonance, defined as the frequency separation between the peak and the dip,^21^ is 27 cm^−1^ and is broader than the molecular resonance.

When the spectrum is measured in the presence of the W(CO)_6_/heptane solution, the resonance enhances the molecular absorption, which appears as a strong dip superimposed on the metasurface resonance. In other words, the coupling resulting from the interaction between the photonic and the molecular resonances enhances the vibrational fingerprint of the analyte.

**Fig. 3:**
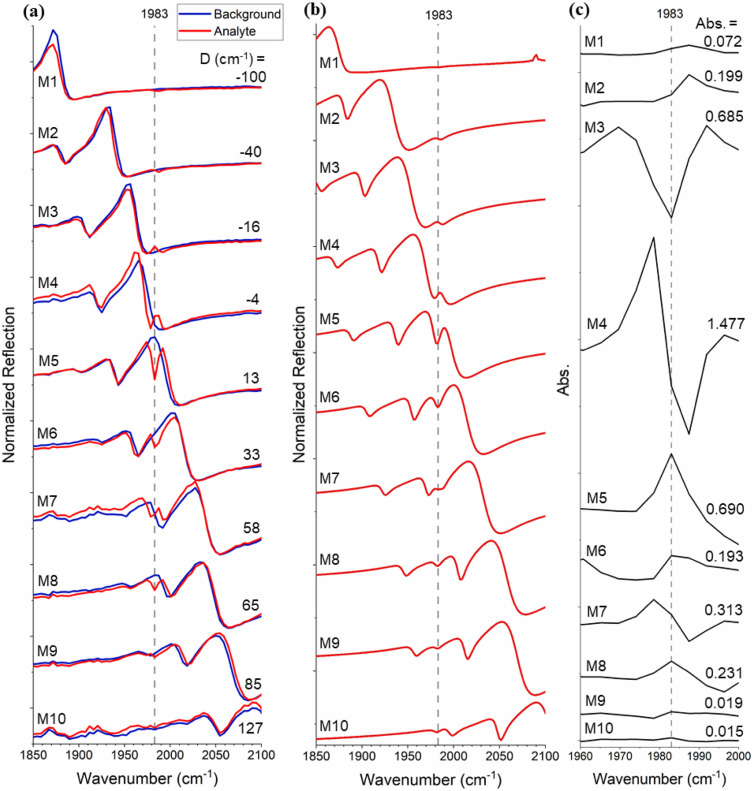
(a) Experimental reflection spectra for metasurfaces with different periods (M1–M10), which demonstrates the tuning of the photonic resonance frequency across the vibrational resonance of the analyte. *D* = *ω*_res_ − *ω*_vib_ denotes the detuning between the photonic and the vibrational resonances. The blue and red graphs represent the spectra in the presence of heptane and W(CO)_6_/heptane solution, respectively. An acquisition time of *t* = 2 s was used for these measurements. (b) Simulated reflection spectra for W(CO)_6_/heptane solution on metasurfaces M1–M10. (c) Metasurface-enhanced absorption spectra of W(CO)_6_ calculated from 
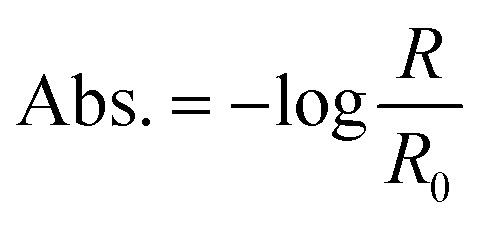
, where *R* and *R*_0_ are the measured reflection of the metasurface with W(CO)_6_/heptane solution and heptane, respectively.

To characterise the signal enhancement further, we tuned the frequency of the Fano resonance (*ω*_res_) with respect to that of the vibrational band (*ω*_vib_), by varying the period of the array. The filling factor (FF), defined as the surface ratio on the metasurface covered by Si to that of the holes, has been kept constant at FF = 0.8. Ten different metasurfaces (M1–M10) were fabricated (see Table S1 in the ESI† for geometric parameters), resulting in a range of resonant frequencies *ω*_res_ (Fig. 3(a)), which were designed to step the resonance across the molecular absorption line (*ω*_vib_ = 1983 cm^−1^). Measurements with heptane and W(CO)_6_/heptane solution are shown by the blue and red traces, respectively. Resonant enhancement of the W(CO)_6_ absorption is clearly observed as the detuning between *ω*_res_ and *ω*_vib_ (*D* = *ω*_res_ − *ω*_vib_, Fig. 3(a)) approaches zero. Notably, not only the size of the absorption feature but also its lineshape varies as a function of detuning. This behaviour is in good agreement with the theory of coupled oscillators and is supported by the simulation results in Fig. 3(b).

In order to calculate the metasurface-enhanced absorption values, we use the absorption equation:1
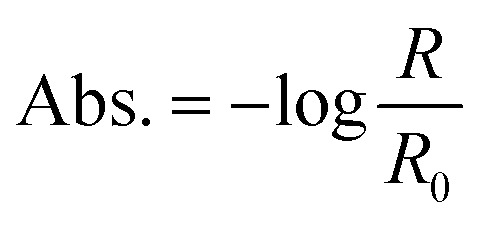
where *R* is the reflection measured in the presence of the absorbing solution and *R*_0_ is the reference reflection, measured in the presence of the solvent only. As is apparent from Fig. 3(c), the SEIRA signals show asymmetric line shapes that can be attributed to the coupling between the molecular vibrations and the metasurface resonance.^2^ The corresponding absorption values, measured from peak to peak of the lineshape,^11^ show how the magnitude of the SEIRA signal varies as a function of detuning. Analysis of Fig. 3(c) shows that the ratio between on-resonance and off-resonance absorption is as much as two orders of magnitude (Abs. increases from 0.015 to 1.477 as |*D*| decreases from 127 to 4 cm^−1^). Furthermore, comparing the highest absorption value achieved by M4 to that of an ATR-FTIR measurement of the same sample (Fig. 2(a)) shows an order of magnitude enhancement.

**Fig. 4:**
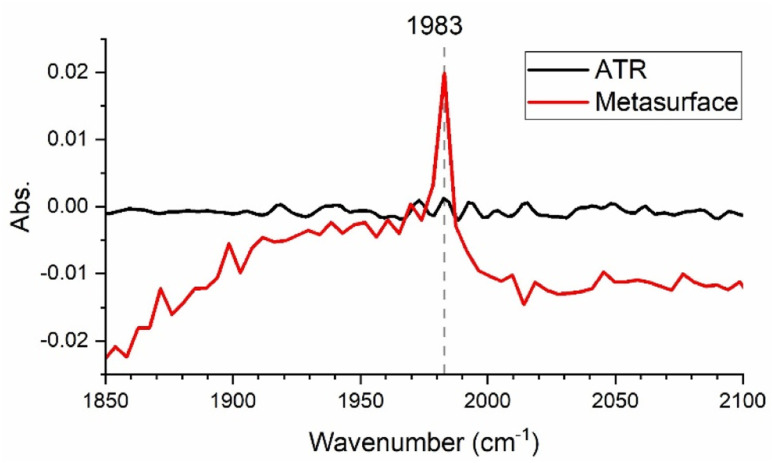
(a) Metasurface-enhanced spectrum (red) for a 0.1 mM W(CO)_6_/heptane solution in comparison to its attenuated total reflection (ATR)-FTIR spectrum.

This enhancement enables us to reach lower limits of detection compared to common spectroscopic methods. Fig. 4 shows the signal resulting from the metasurface-enhanced interaction with the analyte (red trace) for a 0.1 mM W(CO)_6_/heptane solution in comparison to its ATR-FTIR spectrum (black trace). While no absorption line is detectable in the ATR-FTIR spectrum, use of the metasurface leads to a clear signal at 1983 cm^−1^. The complex nature of the spectra in Fig. 3 can be explained by the fact that they describe the interference between the Fano resonance of the metasurface and that of the Lorentzian molecular resonance. In other words, our SEIRA signal originates from the interference of three different resonances: (i) the Fabry–Perot resonance of the waveguide slab, (ii) the Bragg resonance of the periodic array, and (iii) the molecular vibration. Such distorted line shapes for SEIRA signals have previously been reported and are explained by the phase difference between the contributing resonances.^22–24^

### Enhancement of broad vibrational bands

**Fig. 5:**
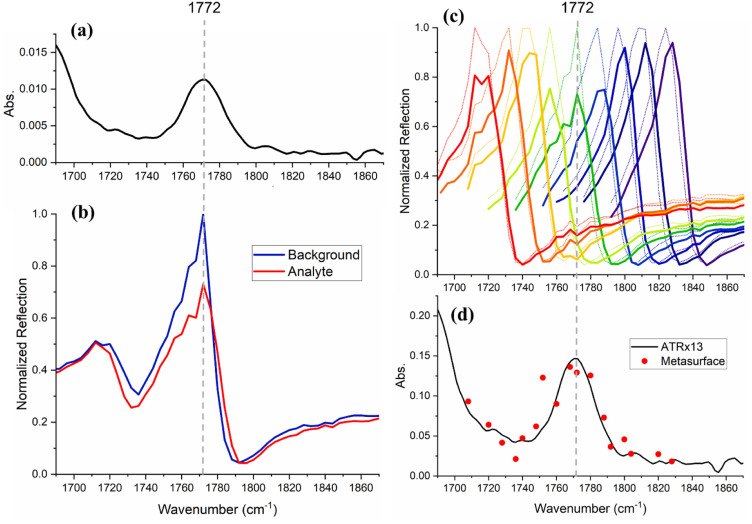
(a) Attenuated total reflection (ATR) spectrum of amoxicillin determined by subtraction of the spectrum of pure DMSO from that of the amoxicillin/DMSO solution. (b) Normalized reflection spectra of the metasurface with DMSO (blue) and amoxicillin/DMSO solution (red). (c) Reflection spectra measured by sweeping the light beam over a chirped metasurface with a varying period that allows for resonance tuning along the vibrational band, in the presence of DMSO (dotted lines) and amoxicillin/DMSO (solid lines). (d) Amoxicillin absorption values calculated from the changes in the reflection of the metasurface resonance peak in the absence/presence of the absorbing analyte (red datapoints) in comparison to the ATR-FTIR spectrum magnified by 13 times (black line).

Next, we show that our method can be extended to analytes which give rise to broader absorption bands, as are commonly found in aqueous media and samples of a biomedical nature. A 60 mM solution of amoxicillin, a commonly prescribed antibiotic, in dimethyl sulfoxide (DMSO) was prepared. The corresponding solvent-subtracted ATR-FTIR spectrum (Fig. 5(a) and S3†) shows an absorption band centred at 1772 cm^−1^ with an FWHM of 33 cm^−1^, which is assigned to a carbonyl stretching vibrational mode. This molecular resonance is broader than that of the metasurface designed for this analyte, which has an FWHM of 20 cm^−1^. Correspondingly, the molecular absorption does not simply form a dip on top of the photonic resonance but shows itself as a change in amplitude, as observed in Fig. 5(b). We are therefore able to reconstruct the entire absorption band using multiple resonances acquired by gradual changes in the periodicity of the metasurface, known as a “chirped” metasurface.^25^ Correspondingly, we fabricated a chirped structure with a period varying from 3.393 to 3.798 (±0.001) μm in 0.25 nm steps. By moving the position of the beam across this chirped metasurface, one can change the period of the structure that interacts with the beam and so determines *ω*_res_ (Fig. 5(c), see also Fig. S4 in the ESI†). As the beam spot is 150 μm, a change of about 10 nm in the period is obtained across the beam spot. The reflection ratio of the amoxicillin/DMSO solution *versus* DMSO was used to generate the spectrum shown with red datapoints in Fig. 5(d). For clarity, only a subgroup of the spectra used for calculating these datapoints are shown in Fig. 5(c). A comparison of these absorption values with those obtained by ATR-FTIR (black trace) demonstrates an enhancement by a factor of 13. We explain the difference between this 13-fold enhancement with respect to ATR and the 100-fold enhancement between resonant and non-resonant absorption described earlier, with the fact that the penetration depth of light is larger in the ATR configuration compared to the non-resonant reflection. The penetration depth (*d*_p_) and effective path length (*d*_e_) for the ATR configuration are calculated (using eqn (S1)–(S5) in the ESI†) to be 1.06 and 2.22 μm, respectively.

## Methods

### Metasurface fabrication

The metasurfaces were fabricated on CaF_2_ substrates (Crystran), which are highly transparent and have a refractive index (*n*) of ∼1.4 in the mid-IR range. A 400 nm thick amorphous Si layer (*n* = ∼2.75) was sputtered onto the substrate using a custom-built pulsed DC magnetron sputterer under Ar flow (40 sccm). Pattern definition was carried out by electron beam (e-beam) lithography (RAITH Voyager®) followed by reactive ion etching (RIE) for structure formation. ARP-13 resist (Allresist GmbH) spin-coated at 2000 rpm for 60 s and baked on a 180 °C hotplate for 10 min was used as the e-beam resist. In addition, a thin layer of AR-PC 5090 (Allresist GmbH) was spin-coated at 2000 rpm for 60 s and baked at 90 °C for 2 min to prevent the charging effect during the e-beam exposure, as the CaF_2_ substrate is non-conducting. An e-beam dose of 180 μC cm^−2^ was used to expose the resist. The sample was then washed in deionized water to remove the charge-dissipating coating. The resist development was performed in xylene for 2 min, followed by a rinse with isopropanol and drying with nitrogen. RIE with a gas mixture of 14.5 sccm CHF_3_ and 12.5 sccm SF_6_, in a chamber pressure of 0.4 mbar was used to transfer the holes pattern to the Si layer. Finally, the resist residues were removed by dipping the chip in warm 1165 solvent (MicroChem), then rinsed in acetone and isopropanol, and dried by nitrogen.

### Spectroscopy

Infrared (IR) transmittance measurements were carried out using a Fourier transform (FT) spectrometer (Bruker, Vertex 70v), with air background. An attenuated total reflection (ATR) unit (Harrick, MVP 2 Series™) with a diamond crystal and 45° incidence angle, was used to collect ATR-FTIR spectra.

The metasurface-enhanced absorption measurements were performed using one third of the output of a Ti-sapphire femtosecond laser (Spectra-Physics Solstice Ace, 800 nm, 6 W, 90 fs, 1 kHz pulse repetition rate) to pump an optical parametric amplifier (OPA, Spectra-Physics TOPAS Prime) equipped with noncollinear difference frequency generation. The resulting mid-IR laser pulses had a duration of ∼150 fs and a bandwidth of ∼200 cm^−1^. The central frequency was tuned to match the molecular resonance of the sample being studied. A parabolic gold mirror was used to focus the beam onto the metasurface from the substrate side with a numerical aperture (NA) of 0.02, at a near-normal incidence angle. The reflected beam was recollimated using the same parabolic mirror, prior to frequency-dispersion and detection using a spectrograph (Horiba Jobin Yvon Ltd, Triax) and a liquid nitrogen-cooled mercury cadmium telluride (MCT) detector (Infrared Associates Inc, MCT-6-64). All spectra were divided by the reflection spectrum of a flat Si layer on the CaF_2_ substrate in the same experimental conditions, to normalize the signal to the laser energy profile and differences in pixel sensitivity. All spectra were then normalized to the maxima of the corresponding spectra in the presence of the solvent.

### Numerical simulations

The numerical calculations were performed using the finite difference time domain (FDTD) method (Ansys/Lumerical), which solves Maxwell's equations in time and the three spatial dimensions. A plane-wave light source was used for the simulations. Periodic boundary conditions in the lateral direction and perfectly matching layers (PML) in the propagation direction of light were applied. Nondispersive refractive indices of 1.4, 2.75, and 1.38 were adopted for CaF_2_, Si, and heptane, respectively. An extinction coefficient of 0.01 was considered for a-Si, while the rest of the materials were assumed to be lossless. The absorption band for W(CO)_6_ was modelled as a Lorentzian oscillator with a centre frequency of 1983 cm^−1^.

## Conclusions

We have demonstrated a resonant photonic platform for surface-enhanced infrared absorption (SEIRA) spectroscopy in dilute solutions. This extends the scope for potential applications of SEIRA methods, which have so far been restricted to thin films and surface-adsorbed molecules. This successful demonstration is based on the use of an all-dielectric silicon metasurface that provides strong light–matter interaction over a micrometre-scale interaction length. Despite the smaller field enhancement in our design compared to what has been previously reported, which corresponds to lower confinement of light, bulk samples and liquid specimens benefit from the extension of the field enhancement over significantly larger volumes offered by our metasurface. We observe an enhancement between resonant and non-resonant absorption by up to two orders of magnitude and an enhancement compared to ATR-FTIR by an order of magnitude. Such an enhancement has enabled us to achieve a limit of detection lower than that of ATR-FTIR. We also show that despite the narrow bandwidth of the metasurface resonance, we can enhance the absorption of a spectrally broad vibrational feature by using a chirped approach whereby the lattice period of the resonant metasurface varies continuously across the sampling area. The reflection configuration enabled by the metasurface approach has the additional benefit of limiting the light path length in the sample, which an elegant solution to the problem of excessive background absorption (*e.g.* due to water) that is otherwise encountered in transmission setups. The ubiquity of solution-phase samples in chemical, industrial and biomolecular applications means that this advance opens up a range of potential analytes. In particular, we envisage the application of this technology to extend the scope of vibrational spectroscopy for the analysis of biofluid samples and for drug metabolism and pharmacokinetics (DMPK) applications as well as providing quality control for the production of biological and biosimilar molecules. The ability to enhance IR absorption and the sensitivity of absorption spectroscopy in general will also be beneficial for applications that use intrinsic molecular vibrational modes as site-specific probes for biomolecular interactions, such as protein–drug binding, which are often hindered by weak probe signals and low biomolecule solubility. The simplicity and mobility of the demonstrated photonic chip facilitate its integration into other systems, such as nonlinear and transient IR spectroscopy as well.

## Data availability

Additional data and supporting information are provided in the ESI.† The dataset related to this article can be obtained from https://doi.org/10.15124/18251cfa-53a9-4180-92a6-416e488e444f.

## Author contributions

SK led the conceptualization, investigation, data curation, analysis and writing of the original draft. DC, BP, and DJS supported the investigation and analysis. NTH and TFK led the supervision, conceptualization, funding acquisition, review and editing of the draft. All authors have given approval to the final version of the manuscript.

## Conflicts of interest

The authors claim no conflicts of interest.

## Supplementary Material

SC-013-D2SC03927C-s001
