# Supplementary material for: Metasurface-enhanced mid-infrared spectroscopy in the liquid phase
Source: Chem Sci. 2022 Oct 21;13(43):12858–64. doi: 10.1039/d2sc03927c (PMC9645393; doi:10.1039/d2sc03927c)
Supplement: SC-013-D2SC03927C-s001 [file SC-013-D2SC03927C-s001.pdf]

Electronic Supplementary Material (ESI) for Chemical Science.
This journal is © The Royal Society of Chemistry 2022

Supplementary Information

Metasurface-enhanced mid-infrared spectroscopy in the liquid phase

Soheila Kharratian[1,2], Donato Conteduca[2], Barbara Procacci[1], Daniel J. Shaw[1], Neil T. Hunt[1], Thomas F. Krauss[2]

1. Department of Chemistry and York Biomedical Institute, University of York, Heslington, York, YO10 5DD, UK

2. School of Physics, Engineering and Technology and York Biomedical Research Institute, University of York, Heslington, York, YO10 5DD, UK

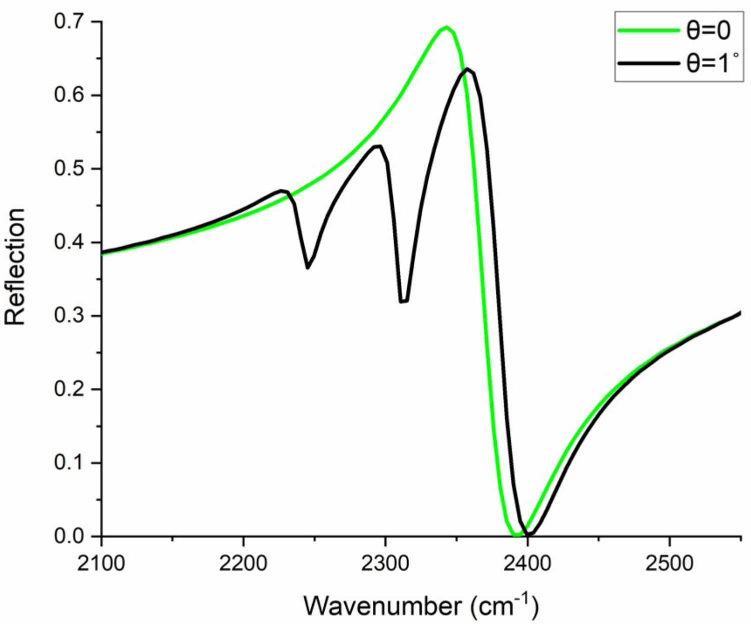

Figure S1. The optical response of the metasurface, simulated using the finite-difference time-domain (FDTD) method, for normal (green) and near-normal (black) incidence. θ is the incidence angle measured from the surface normal. We have used a small incidence angle of about 1˚ in our experiments to be close to a normal incidence while avoiding the practical complications of using a beam-splitter.

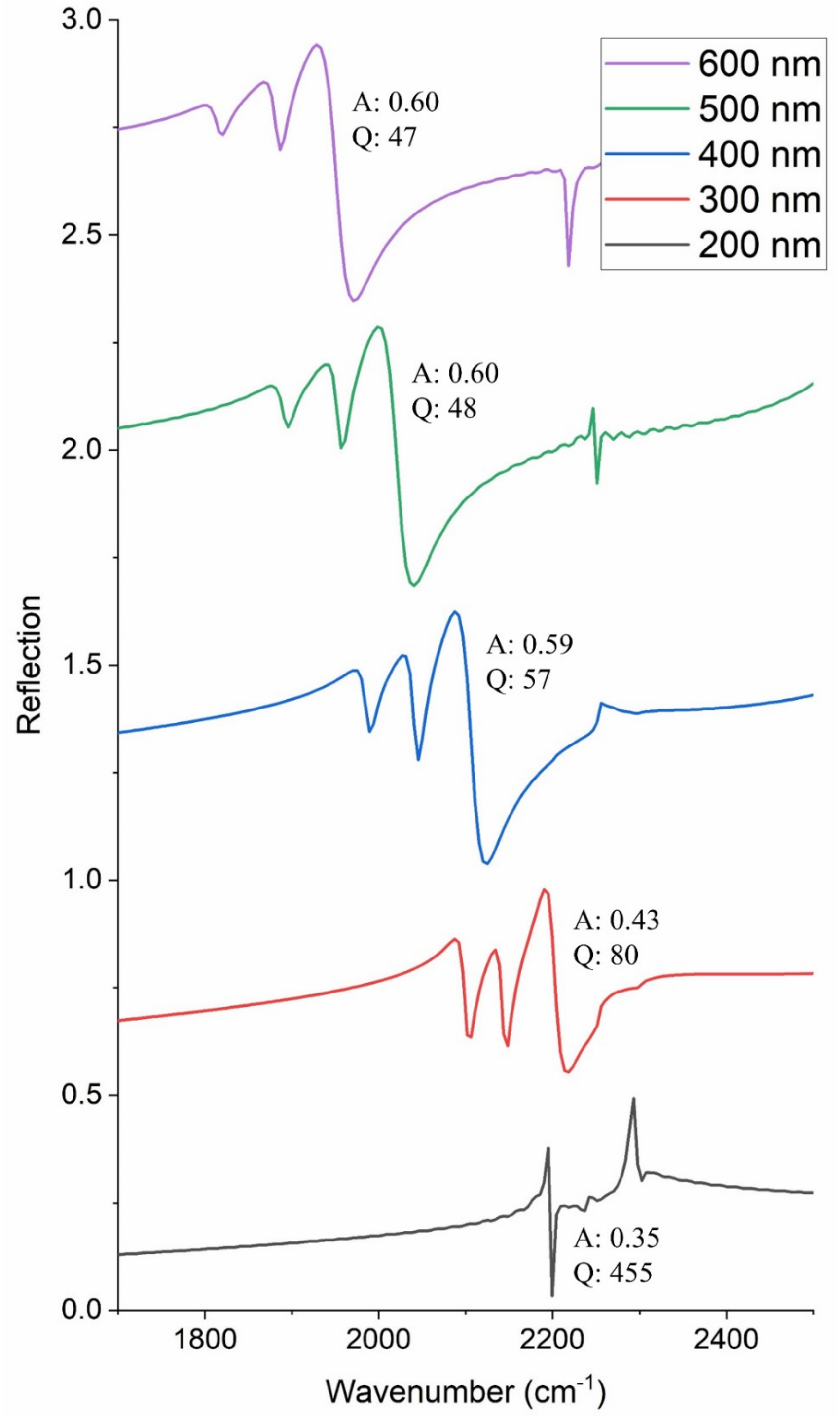

Figure S2. Simulated reflection spectra for metasurfaces with different thicknesses of the silicon slab. The graphs are offset for clarity purposes. The resonance amplitude and quality factor are shown by A and Q, respectively. Despite the decreasing trend of the Q-factor by increasing the silicon thickness, a significant improvement in the resonance amplitude is observed by increasing the thickness up to 400 nm. Thicker Si layers do not result in better performance of the metasurface while making the fabrication more difficult and costly. Hence, 400 nm was chosen as the optimal thickness for the metasurfaces used in this study.

Table S1. List of different metasurfaces fabricated for tuning the photonic resonance over the vibrational band of $W(CO)_6$. All the metasurfaces are realized on a 400-nm thick amorphous Si deposited on CaF_2 substrates. The filling factor (surface ratio on the metasurface covered by Si to that of the holes) is kept constant at 0.8, which defines the hole radius based on the period of the structure.

Metasurface	Period (μm)	Holes radius (nm)
**M1**	3.415	862
**M2**	3.289	830
**M3**	3.248	820
**M4**	3.209	810
**M5**	3.171	800
**M6**	3.133	790
**M7**	3.097	781
**M8**	3.061	772
**M9**	3.026	763
**M10**	2.959	747

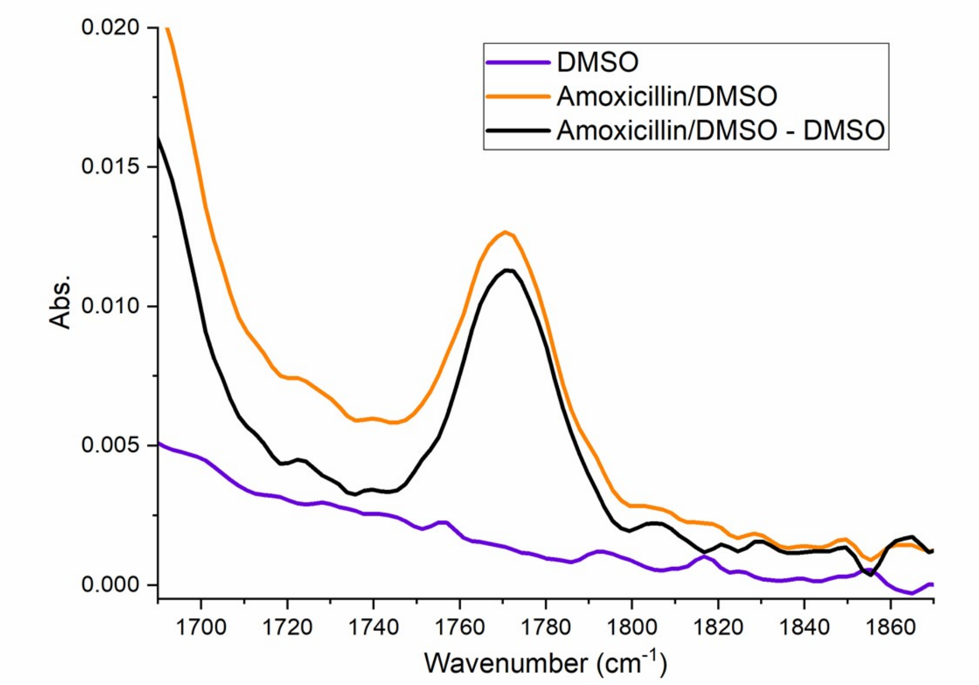

Figure S3. Attenuated total reflection (ATR)-FTIR spectra for pure DMSO (violet) as the solvent and 25 mg/ml Amoxicillin/DMSO solution (orange), and the solvent-subtracted amoxicillin spectrum (black).

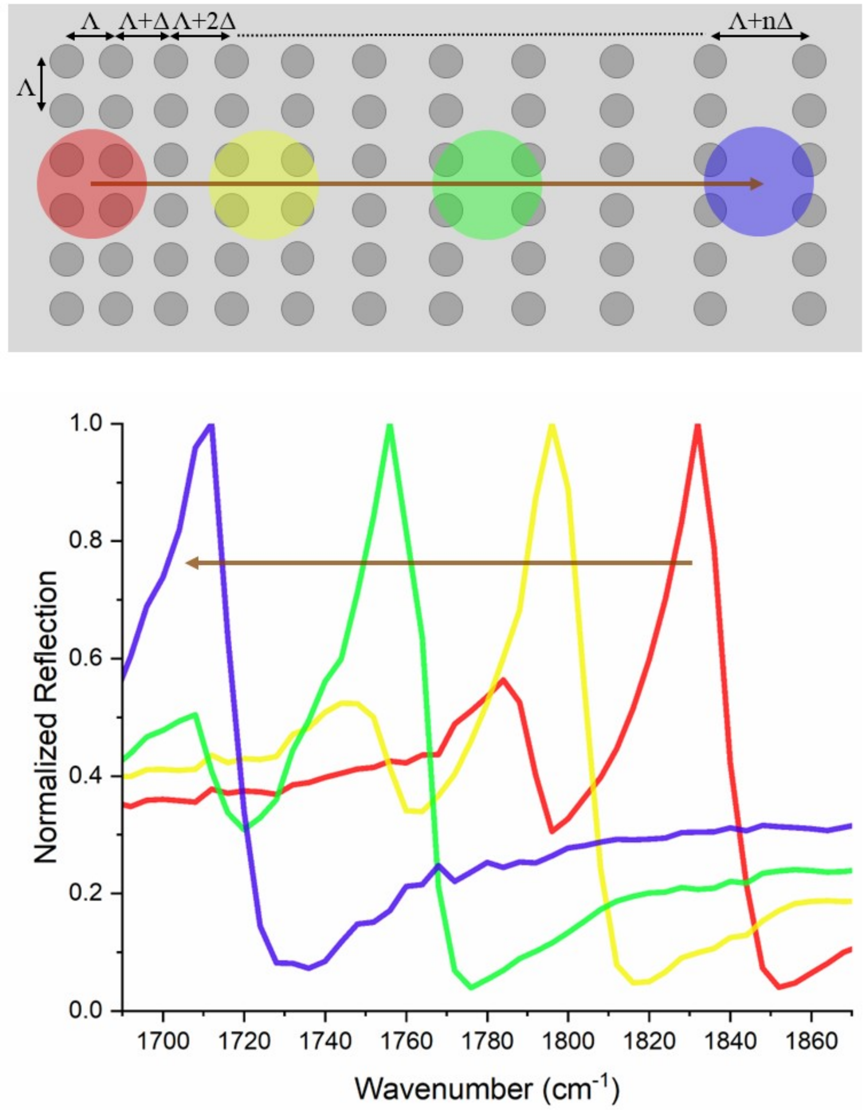

Figure S4. The chirped metasurface has an array of holes with a constant radius and a gradually changing period. The metasurface that we fabricated for studying the absorption band of amoxicillin at 1772 cm^{-1} has a hole radius of 907 nm and a period changing from Λ= 3.393 to 3.798 µm, with change steps of Δ= 0.25 nm. By gradually moving the IR beam towards the longer period (as shown by the brown arrow on the top panel), the Fano resonance moves towards lower frequencies (as observed in the bottom panel), which enables tuning of the resonance along the vibrational band for broadband surface-enhanced spectroscopy. The diameter of the beam spot is 150 µm, covering a period change of about 10 nm in one step.

Penetration depth and effective path length in attenuated total reflection (ATR) configuration

The penetration depth of the evanescent field in ATR configuration is calculated by:

$$d_p = \frac{\lambda}{2\pi(sin^2\theta - n_{21}{}^2)^{1/2}}$$

(Equation S1)

$$n_{21} = \frac{n_2}{n_1}$$

(Equation S2)

where λ and θ are the wavelength and incidence angle, and n_2 and n_1 are the refractive indices of the analyte and the crystal, respectively.

The relative effective path length for perpendicular and parallel polarizations are given by:

$$d_{e\perp} = \frac{\lambda\, n_{21}\cos\theta}{\pi(1 - n_{21}{}^2)(sin^2\theta - n_{21}{}^2)^{1/2}}$$

(Equation S3)

$$d_{e\parallel} = \frac{\lambda\, n_{21}\cos\theta\,(2sin^2\theta - n_{21}{}^2)}{\pi\left(1 - n_{21}{}^2\right)[\left(1 + n_{21}{}^2\right)sin^2\theta - n_{21}{}^2](sin^2\theta - n_{21}{}^2)^{1/2}}$$

(Equation S4)

For unpolarized light, the effective path length is the average of those calculated for s- and p-polarizations[1,2]:

$$d_e = \frac{d_{e\perp} + d_{e\parallel}}{2}$$

(Equation S5)

References

1 N. J. Harrick and F. K. Pre, *Appl. Opt.*, 1966, **5**, 1739–1743.

2 L. A. Averett and P. R. Griffiths, *Anal. Chem.*, 2008, **80**, 3045–3049.
